# Development of a Safety Management Web Tool for Horse Stables

**DOI:** 10.3390/ani5040402

**Published:** 2015-11-12

**Authors:** Jarkko Leppälä, Christina Lunner Kolstrup, Stefan Pinzke, Risto Rautiainen, Markku Saastamoinen, Susanna Särkijärvi

**Affiliations:** 1 Natural Resources Institute Finland (Luke), Latokartanonkaari 9, 00790 Helsinki, Finland; 2 Swedish University of Agricultural Sciences (SLU), 23053 Alnarp, Sweden; E-Mails: christina.kolstrup@slu.se (C.L.K.); stefan.pinzke@slu.se (S.P.); 3 University of Nebraska Medical Center, Omaha, NE 68198, USA; E-Mail: risto.rautiainen@luke.fi; 4 Natural Resources Institute Finland (Luke), Opistontie 10 a 1, 32100 Ypäjä, Finland; E-Mails: markku.saastamoinen@luke.fi (M.S.); susanna.sarkijarvi@luke.fi (S.S.)

**Keywords:** horse stable, safety, management, web tool

## Abstract

**Simple Summary:**

A new web tool for equine activities, InnoHorse, was developed to support horse stable managers in business, safety, pasture and manure management. The aim of the safety section of the web tool was to raise awareness of safety issues in daily horse stable activities. This section contains a safety checklist, stable safety map and good practices to support human health and horse welfare and to prevent injuries in horse-related activities. Reviews of the literature and statistics, empirical horse stable case studies, expert panel workshops and stakeholder interviews were utilized in designing the web tool.

**Abstract:**

Managing a horse stable involves risks, which can have serious consequences for the stable, employees, clients, visitors and horses. Existing industrial or farm production risk management tools are not directly applicable to horse stables and they need to be adapted for use by managers of different types of stables. As a part of the InnoEquine project, an innovative web tool, InnoHorse, was developed to support horse stable managers in business, safety, pasture and manure management. A literature review, empirical horse stable case studies, expert panel workshops and stakeholder interviews were carried out to support the design. The InnoHorse web tool includes a safety section containing a horse stable safety map, stable safety checklists, and examples of good practices in stable safety, horse handling and rescue planning. This new horse stable safety management tool can also help in organizing work processes in horse stables in general.

## 1. Introduction

**Figure 1:**
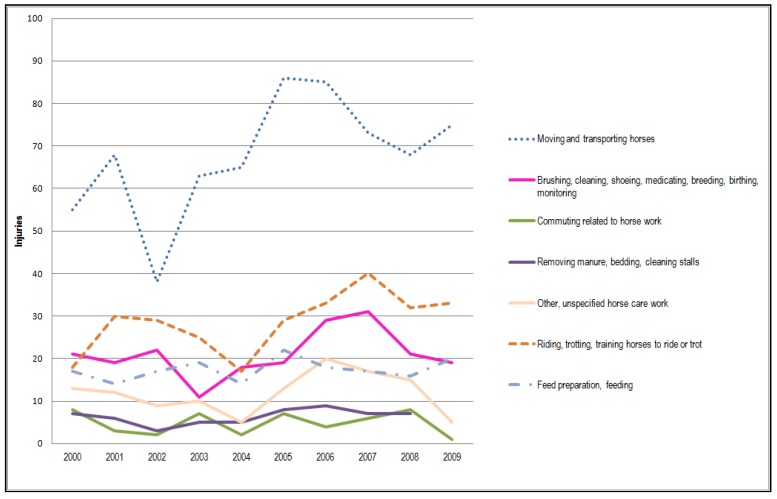
Statistics from Farmers Social Insurance Institution, Finland concerning occupational injuries among equine entrepreneurs in horse-related activities during 1990–2009 [7,8].

**Figure 2:**
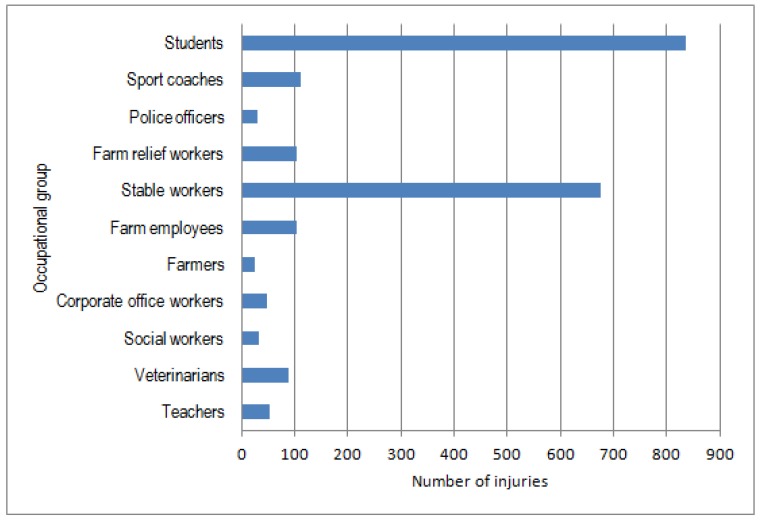
Statutory accident insurance (TVL) statistics from Finland concerning the total numbers of injuries to horse stable workers and other persons related to occupational horse activities during 2003–2010 [12].

The equine sector has grown strongly in recent years in many European countries [1,2]. For example, the number of horses has almost doubled in Finland during the past thirty years [3]. In Sweden, in turn, the number of horses per capita is the largest in Europe [1]. The equine sector, with diverse activities, provides an attractive lifestyle and rewarding experiences, but to be successful, good safety management skills and practices are needed. A problem is that safety risks and injuries are high in many horse-related activities. Without awareness of the possible risks and proper knowledge and skills of horsemanship, people engaged in horse-related activities will be exposed to many safety risks that may have serious consequences [4,5,6]. For example, in Finland, approximately 170 injuries occur per year among horse entrepreneurs and about 300 incidents among persons in other professional sectors working with horses (such as students, farmers, relief workers, veterinarians) (Figure 1). In other professional sectors, most of the injuries involve students and stable workers (Figure 2) [6,7].

Leisure time injury statistics are still largely unknown [6,7,8]. In Sweden, the exact number of horse-related injuries is uncertain because of underreporting. Nevertheless, in 2012, nearly 12,900 persons went to an emergency centre after being injured in riding accidents or other activities related to horse handling. Nearly nine out of ten injured persons were females and 40% were children younger than 18 years of age. Injuries were more frequent among girls aged 10 to 19 years compared to other age groups [9,10]. According to the statistics of the Finnish Farmers’ Social Insurance Institution, Mela [8,11], almost 35% of human injuries in horse activities have been serious incidents that have resulted in over 30 days of sick leave.

Consequently, good risk management tools and safety practices are needed in the equine business and horse activities. A project titled InnoEquine financed by the EU Central Baltic programme was carried out in Finland, Sweden, Latvia and Estonia during 2010–2013. The overall aim of the project was to enhance the competitiveness of equine entrepreneurs in the Baltic region and to promote sustainable management in the equine sector. As one of the results of the InnoEquine project, the InnoHorse safety web tool for horse stables was developed in order to identify new practical solutions for risk and safety management [7].

## 2. Data and Methods

The InnoEquine project was carried out jointly by MTT Agrifood Research Finland (presently the Natural Resources Institute Finland (Luke), the Swedish University of Agricultural Sciences (SLU) and the Latvian University of Agriculture (LTU). A specific aim of the project was to develop a web tool providing good practices in environmental, human safety and horse welfare activities for the equine sector. This paper focuses on the design and development of the safety web tool in the project. The purpose of this safety web tool was to provide knowledge and practical tools to prevent injury incidents and occupational diseases in the equine sector.

**Figure 3:**
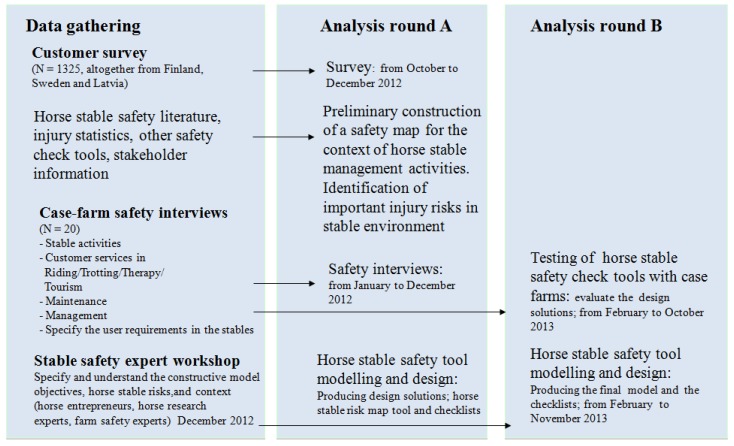
The design, methods, processes, analysis tasks and timetables for preparing the InnoHorse safety web tool [13].

The design, methods and processes of the stable safety web tool, as well as the tasks and timetables are presented in Figure 3 [13]. Basic information related to management activities and safety needs in horse stables in Finland and Sweden were gathered through a customer survey (*N* = 1325) [14]. An expert workshop on horse stable safety was held (at MTT) in December 2012 to assist with the design of the web tool. The workshop identified major risk categories and specific risks associated with horse stable activities and functions. The participants (*n* = 10) included horse sector experts, horse farm managers, farm safety, security, and risk management experts from Finland and Sweden, and Innoequine project representatives. The workshop utilized existing Farm Risk Map [15] tools and procedures as the starting point. Participants used a wallpaper technique, writing their ideas on wall notes, based on their perspectives and experiences. The identified horse stable risks and themes were documented, photographed, grouped, and arranged under redefined risk categories.

Ten case studies on horse farms were analyzed for the safety section both in Finland and in Sweden. The case farms included enterprises in different size categories and different types of stables. All interviews were documented.

Information on current legislation, standards, and various horse safety and management guidebooks was gathered from the participating countries. These data were analyzed and integrated to produce the background for safety risk identification in horse stable activities. In these cases, we used broader recommendations based on standards, research, International Labour Organization (ILO) recommendations or EU directives [7,16]. The comprehensive collection of photos and documentation to illustrate good practices was gathered through numerous farm visits, equine fairs and equine companies in the three participating countries.

In addition, we conducted literature reviews, analyses of insurance claims involving horse injuries, Internet searches, comparison of various farm safety risk tools, discussions with equine organizations and stakeholders, and interviews and visits to horse farms. Results from these investigations have been reported elsewhere [13,16,17].

All collected data were processed in an iterative development cycle and constructive analysis, which included content analysis and designing the preliminary horse stable safety checklists and risk map. The iterative development cycle process is a standardized method that uses control stages to analyze data before going further in the interactive system design process. The process includes stages like (1) understand and specify context of use; (2) specify user requirements; (3) produce design solutions to meet these requirements; (4) evaluate design against requirements [18,19]. This method is widely used in technical and management sciences. Constructive research method is a problem solving method for construction and testing of models to reach a certain objective in a system or context [20]. The final content of the safety web tool was tested during 2013 prior to making it public online (Figure 3). Feedback and data from the safety web tool were gathered by case farmer phone interviews and by the email. The final content was edited by MTT for the InnoHorse web site in English and Finnish and thereafter also translated into Swedish and Latvian [7,16].

## 3. Results and Discussion

### 3.1. The Innohorse Web Tool

In this project, the new InnoHorse web tool was designed to assist in horse stable management practices. The web tool was published by the National Equine Competence Association of Finland (Hippolis). The InnoHorse web site includes management tools for horse stable activities such as safety, manure, pasture, and innovation management. The web tool has been published in English, Finnish, Swedish and Latvian. The layout and information are the same in the different language versions but with minor country-specific differences [7].

**Figure 4:**
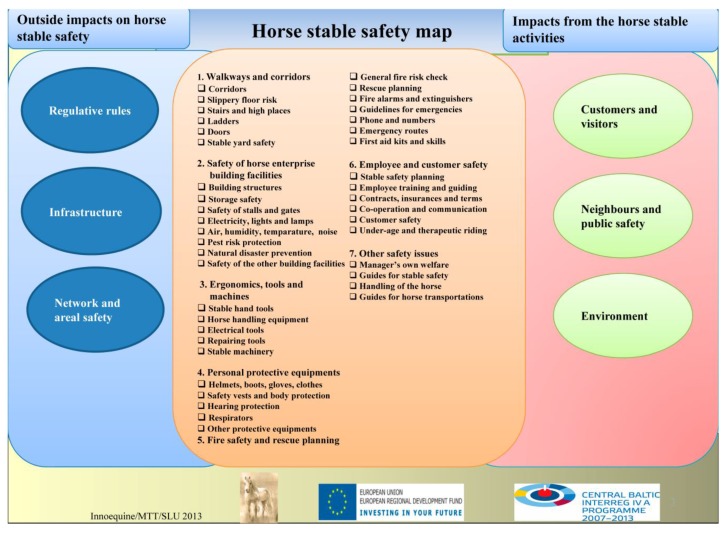
The horse stable safety map.

All major areas of the web tool include an introduction section followed by good practices applied to the particular horse stable management sections. The horse stable safety management section in InnoHorse provides safety information, safety checklists, safety management practices and a horse stable safety map, which is presented in Figure 4. All tools were designed to improve health, reduce safety risks and prevent injuries among horse stable workers, stable managers, clients, visitors and horses.

### 3.2. Safety Section of the Web Tool

The section on safety provides information for horse stable managers in the Baltic Sea region related to various aspects of safety and health management. The web pages of the InnoHorse safety section include a stable safety checklists, one-page horse stable safety map, and examples of good practices for stable safety, horse handling and rescue planning.

The safety web site content begins by introducing injury statistics, mainly in Finland, and the characteristics of horse-related injuries in the equine sector. In Finland, injury statistics for all farm owners, including horse farm owners, are maintained by the Farmers’ Social Insurance Institution (Mela). Statistics on horse-related injuries in the other participating project member countries are scarce. The Finnish statistics indicate that the risk of injuries is nearly three times higher on horse farms compared to grain farms [8].

### 3.3. Safety Checklists and the Horse Stable Safety Map

Systematic risk checklists are practical self-assessment tools for identifying and managing risks in various tasks [15,21,22]. Based on the findings and information from statistics, the stable safety expert workshop, horse farmer interviews and literature search, we developed a stable safety checklist, which addresses potential safety issues in walkways and corridors, built facilities, work ergonomics, equipment and machinery (Figure 4). Personal protective equipment (PPE), fire safety and rescue planning, employee and client safety, as well as some other safety issues such as the transportation of horses and horsemanship skills were also included. The questions in the safety checklists enable the screening of possible risk sources or factors with the potential to cause injuries in and around the stable facilities and around horses. The respondents were asked to estimate whether particular working conditions or activities in a stable were in order. The checklist also includes some guidance or recommendations for reducing potential safety risks. The differences between countries or regions in legislation and safety activities pose a challenge in integrating risk management information in a single safety tool. The horse stable safety map introduces the content of the safety web tool as a one page figure (Figure 4). The idea is the same as in the Farm Risk Map, which was previously designed in Finland by MTT and the Technical Research Centre of Finland (VTT) [15].

### 3.4. Good Practices

**Figure 5:**
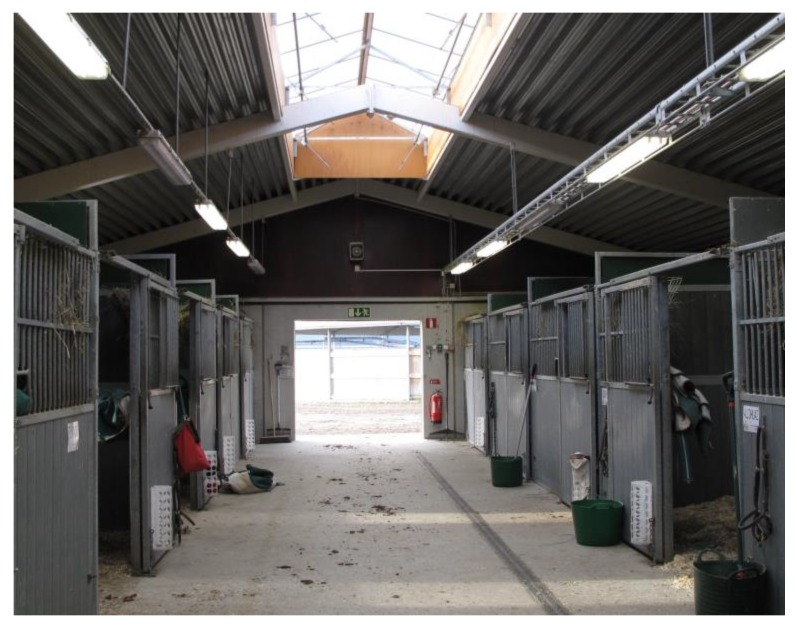
Spacious and bright corridors are important. An example of good safety management practice in horse facilities presented in the InnoHorse web tool. © Christina Lunner Kolstrup.

The section on safety management introduces good practices for persons working or visiting horse stables and riding facilities. According to Mela statistics, a large number of injuries and accidents occur when moving and transporting horses (Figure 1). This is why it is important to have spacious and well-lit corridors with sufficiently wide doorways and sliding stable doors for safe passage with the horse, as well as good ventilation and natural light used together with electric lighting to provide a good work environment for horses and people (Figure 5). The Good Practices section was designed in line with the safety checklist questions, providing further information on stable safety management activities. The section contains information, practical tools, illustrative photos and figures, and examples of good safety practices for horse stable safety management. Overall, practical and efficient stable safety tools can be useful management aids for horse stables.

**Figure 6:**
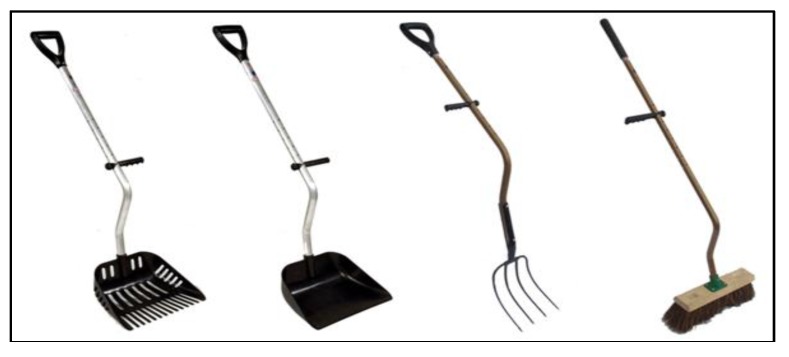
Ergonomically designed long-shafted tools. Reproduced with permission by Lite-Lift Ltd. (http://www.lite-lift.com/, 23 June 2015).

The stable safety management section in the InnoHorse web tool presents practices and guidelines including safety aspects related to stable work, ergonomics, buildings, equipment and machinery use; all important safety factors in the stable work environment. Musculoskeletal disorders and ergonomic problems are very common in horse stables [23]. For example, traditional hand tools are not always adapted for the users, which increases the risk of musculoskeletal disorders in the upper extremities and lower back. Bent shafts in some hand tools help create a more upright posture for the back (Figure 6).

Good handles provide better grip and lightweight tools reduce the workload. Ergonomic tools reduce the workload and the risk of upper limb and lower back musculoskeletal problems. The cleaning of stalls, manure transport and feeding of horses are typical routines. They are time consuming as well as physically demanding work tasks in a stable that require special attention. Feeding and the handling of feeds takes about five to seven minutes per horse per day, and the cleaning of stalls (mucking, replacement of bedding materials) takes approximately 10 minutes per horse daily if no machinery is used [24,25]. Good working clothes, proper equipment and the use of personal protective equipment (PPE), combined with good working conditions, form a good basis for an improved safety culture in horse stables.

### 3.5. Case Study Interviews

The horse stable safety map content was tested with the case farmers. All case farmers were also horse stable managers. The case farmers thought that the horse stable safety map was a comprehensive safety management list for the stable management. They could not find any missing safety management areas that should be added to the horse stable safety map. The structure of the map worked well also during the actual stable visit and stable safety check. It is possible that the case farmers were more interested in safety management than stable managers on average. Their stables were in good condition and well managed. Yet some minor shortcomings were found in almost all stables during the safety check. Most of the shortcomings concerned slippery areas (winter time) on corridors, uneven walkways, lack of lights in the stable and the stable yard, and the farmer’s own welfare. After the safety check the case farmers made several safety improvements in their stable and safety management practices. They had started to use rubber mats on the corridors, improve lighting and ergonomics in the stalls, and they started to think more about their own welfare. They tried to find free time to rest or have a vacation.

According to the interviewed horse stable managers, the most challenging task is to improve people’s safety skills and to get them to behave in a safe manner. Thus, the web tool includes management information on activities such as fire safety and rescue skills, customer and worker safety management, and other behavioral safety issues such as examples of good horsemanship and the importance of rules in stable safety. It is known that poor safety habits are easy to adopt in organizations, so an initial and essential management task is to show and train workers in safe working habits and communicate why safety is important [26]. Thus, the implementation of good safety practices needs to be easy and understandable, and every worker needs to be trained beginning from the first day in a stable. The stable manager’s own self-commitment to safe behavior in stable work is also important as a role model for employees and consumers.

## 4. Conclusions

The differences between countries in legislation and safety activities pose a challenge in designing safety or environmental management guidelines. For this reason, some recommendations are provided on a general level. However, some standards, research studies or directives may help stable managers in acquiring more practical information. Another challenge is human safety behaviour in the horse stable environment. Without good management, poor safety habits may spread in the organization. Thus, the application of good safety practices needs to be as easy as possible and every worker needs to be trained beginning from the first day in the stable. The stable manager’s own commitment to safe behaviour in stable work is also important.

The InnoHorse safety web tool aims to help in organizing and managing safety activities in horse stables and facilities. This tool provides a practical context model for identifying risks in horse stable activities. It contains physical and behavioral risks, which are listed in a compact horse stable safety map. This holistic approach provides a new comprehensive model for risk identification and risk management for the equine sector. The horse stable safety map and other safety check tools in the Innohorse web tool may not solve all the safety problems in horse stables, but hopefully they help some horse stable managers to improve their stable safety management. It is intended to provide tools for the equine sector to inspire, motivate and encourage people to act and behave more safely around horses in order to prevent horse-related injuries.
